# Systematic evaluation of the association between hemoglobin levels and metabolic profile implicates beneficial effects of hypoxia

**DOI:** 10.1126/sciadv.abi4822

**Published:** 2021-07-14

**Authors:** Juha Auvinen, Joona Tapio, Ville Karhunen, Johannes Kettunen, Raisa Serpi, Elitsa Y. Dimova, Dipender Gill, Pasi Soininen, Tuija Tammelin, Juha Mykkänen, Katri Puukka, Mika Kähönen, Emma Raitoharju, Terho Lehtimäki, Mika Ala-Korpela, Olli T. Raitakari, Sirkka Keinänen-Kiukaanniemi, Marjo-Riitta Järvelin, Peppi Koivunen

**Affiliations:** 1 Faculty of Medicine, Center for Life Course Health Research, University of Oulu, 90014 Oulu, Finland.; 2 Medical Research Center, Oulu University Hospital and University of Oulu, 90220 Oulu, Finland.; 3 Biocenter Oulu, 90014 Oulu, Finland.; 4 Faculty of Biochemistry and Molecular Medicine, Oulu Center for Cell-Matrix Research, University of Oulu, 90014 Oulu, Finland.; 5 Department of Epidemiology and Biostatistics, School of Public Health, Imperial College London, Norfolk Place, W2 1PG London, UK.; 6 Research Unit of Mathematical Sciences, University of Oulu, 90014 Oulu, Finland; 7 Computational Medicine, Faculty of Medicine, University of Oulu, 90014 Oulu, Finland.; 8 NMR Metabolomics Laboratory, School of Pharmacy, University of Eastern Finland, 70210 Kuopio, Finland.; 9 Clinical Pharmacology and Therapeutics Section, Institute of Medical and Biomedical Education and Institute for Infection and Immunity, St George’s, University of London, London, UK.; 10 LIKES Research Center for Physical Activity and Health, 40700 Jyväskylä, Finland.; 11 Research Centre of Applied and Preventive Cardiovascular Medicine, University of Turku, 20520 Turku, Finland.; 12 Centre for Population Health Research, University of Turku and Turku University Hospital, Turku, Finland.; 13 NordLab Oulu, Medical Research Center Oulu, Oulu University Hospital and Department of Clinical Chemistry, University of Oulu, 90014 Oulu, Finland.; 14 Department of Clinical Physiology, Tampere University Hospital and Faculty of Medicine and Health Technology, Tampere University, Tampere, Finland.; 15 Department of Clinical Chemistry, Fimlab Laboratories and Faculty of Medicine and Health Technology, Finnish Cardiovascular Research Center - Tampere, Tampere University, Tampere, Finland.; 16 Department of Clinical Physiology and Nuclear Medicine, Turku University Hospital, 20520 Turku, Finland.; 17 Unit of Primary Care, Oulu University Hospital, 90220 Oulu, Finland.; 18 Department of Epidemiology and Biostatistics, MRC-PHE Centre for Environment and Health, School of Public Health, Imperial College London, Norfolk Place, London W2 1PG, UK.; 19 Department of Life Sciences, College of Health and Life Sciences, Brunel University London, Uxbridge, Middlesex UB8 3PH, UK.

## Abstract

Activation of the hypoxia-inducible factor (HIF) pathway reprograms energy metabolism. Hemoglobin (Hb) is the main carrier of oxygen. Using its normal variation as a surrogate measure for hypoxia, we explored whether lower Hb levels could lead to healthier metabolic profiles in mice and humans (*n* = 7175) and used Mendelian randomization (MR) to evaluate potential causality (*n* = 173,480). The results showed evidence for lower Hb levels being associated with lower body mass index, better glucose tolerance and other metabolic profiles, lower inflammatory load, and blood pressure. Expression of the key HIF target genes *SLC2A4* and *Slc2a1* in skeletal muscle and adipose tissue, respectively, associated with systolic blood pressure in MR analyses and body weight, liver weight, and adiposity in mice. Last, manipulation of murine Hb levels mediated changes to key metabolic parameters. In conclusion, low-end normal Hb levels may be favorable for metabolic health involving mild chronic activation of the HIF response.

## Teaser

Low-end normal hemoglobin levels, which carry less oxygen, are associated with lower body weight and healthier metabolism.

## INTRODUCTION

Hemoglobin (Hb), an iron-containing metalloprotein in red blood cells, is the main carrier of oxygen. Hb levels are regulated genetically and environmentally, and they vary by sex, ethnicity, age, and altitude (*1*, *2*). Individuals’ Hb levels during adult life are, however, very stable and used, for example, in the athlete’s biological passport to detect blood doping (*3*). Hb levels directly affect arterial oxygen concentration and thereby tissue oxygenation (*4*, *5*). In general, high-end Hb levels within the normal range are considered beneficial for health (*2*). When tissues encounter reduced oxygen levels, then as a key transcriptional response, the hypoxia-inducible factor (HIF) becomes stabilized. HIF up-regulates genes that induce oxygen delivery and reduce its usage, such as those regulating erythropoiesis and oxidative energy metabolism, respectively (*6*). The stability of the HIFα subunits is governed by three HIF prolyl 4-hydroxylases (HIF-P4Hs; also known as PHDs and EGLNs), which require oxygen for catalysis and target HIFα for destruction in normoxia (*6*). Three HIFα subunits exist, of which HIF1α and HIF2α are the most studied (*6*). Inhibition of HIF-P4Hs and the following activation of the HIF response have been shown to protect mice from obesity, metabolic dysfunction, and associated diseases (*7*–*10*). The evidence in human data is largely lacking.

**Fig. 1:**
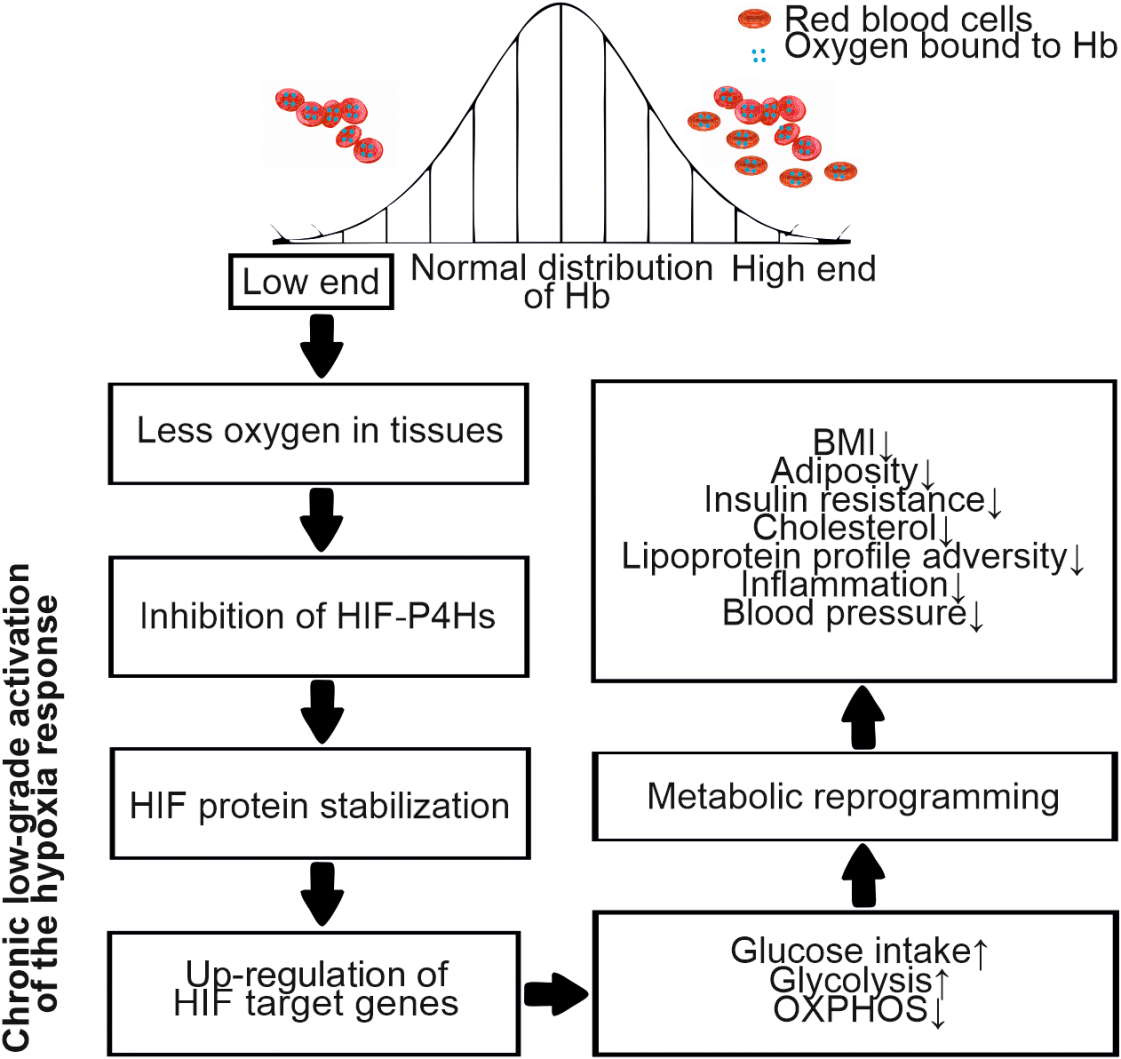
Study hypothesis associating lower Hb levels with better metabolic health. HIF, hypoxia-inducible factor; HIF-P4H, HIF prolyl 4-hydroxylase; OXPHOS, oxidative phosphorylation.

To evaluate whether the HIF response is beneficial for metabolic health in humans, we used the normal variation in Hb levels as a surrogate measure for oxygenation status. We hypothesized that lower Hb levels were hypoxic and could result via HIF-P4H inhibition to HIF-mediated reprogramming of energy metabolism and better metabolic health (Fig. 1). To test this, we used an innovative and multistep systematic and longitudinal approach where we first (i) evaluated the association of Hb levels with key metabolic parameters in mice and then (ii) examined its associations with more than 170 anthropometric and metabolic parameters in humans in cross-sectional and longitudinal settings in two large population-based, sea-level cohorts from Finland. Furthermore, we (iii) applied Mendelian randomization (MR) analysis to explore potential causality of both Hb levels and HIF pathway target gene expression on metabolic traits. In the MR paradigm, the use of genetic variants randomly allocated at conception to proxy the effect of an exposure can help overcome the confounding and reverse causation that hinders causal inference in traditional observational study designs. Last, we (iv) performed a venesection intervention study in mice to evaluate the effect of Hb level manipulation on body weight, glucose, and lipid metabolism.

## RESULTS

### Hb levels associate with anthropometric measures in mice and humans

**Fig. 2:**
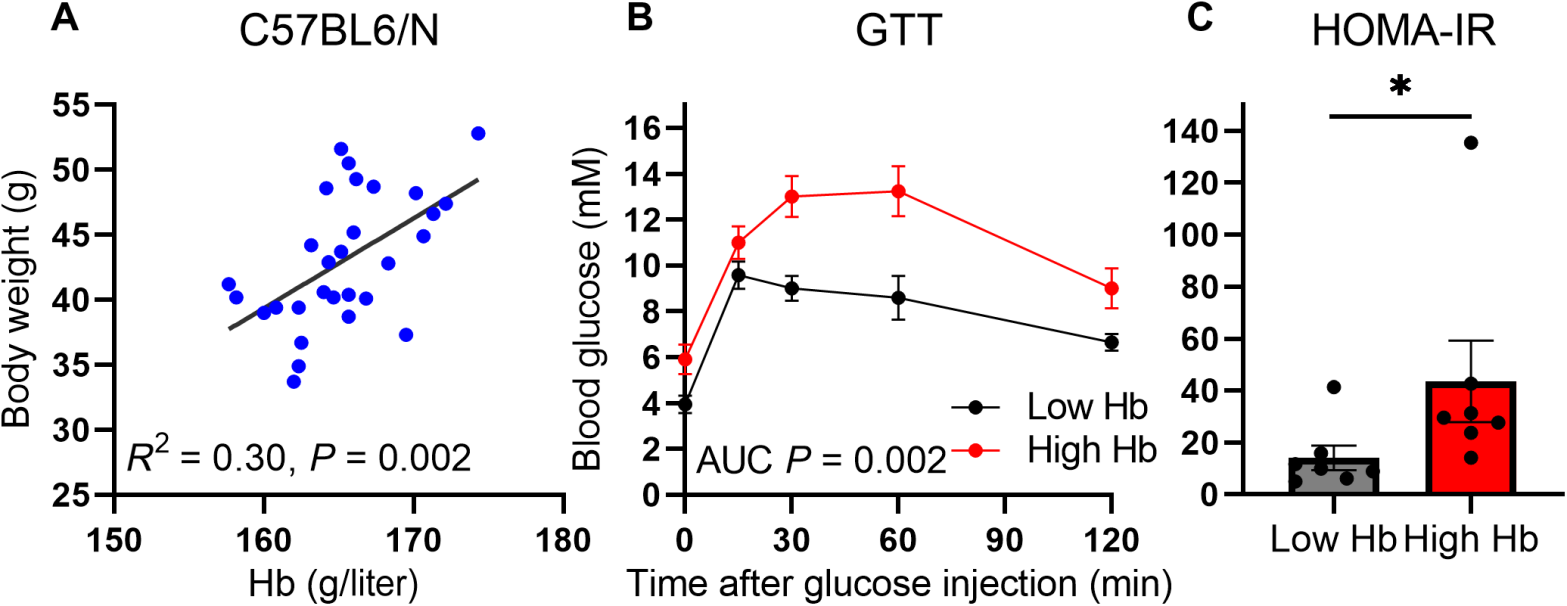
Association of Hb levels with body weight, glucose tolerance, and insulin resistance in mice. (**A**) Association of Hb levels with body weight in 1-year-old C57Bl/6 male mice (*n* = 29). (**B** and **C**) Association of the highest quartile (High Hb) and the lowest quartile (Low Hb) of Hb levels with GTT area under the curve (AUC) and HOMA-IR scores, respectively, in the 1-year-old C57Bl/6 male mice (C). The data in (B) and (C) are means ± SEM. *P < 0.05.

First, we evaluated the potential association of Hb levels with key metabolic parameters in C57Bl/6 mice and found a positive association between Hb levels and body weight at 1 year of age (Fig. 2A). Lower Hb levels associated with more favorable glucose tolerance in a glucose tolerance test (GTT) and with lower homeostatic model assessment of insulin resistance (HOMA-IR) scores (Fig. 2, B and C), suggesting that Hb levels may influence metabolic health.

**Fig. 3:**
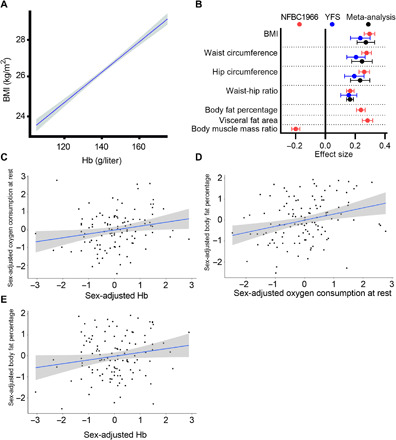
Association of Hb levels with key anthropometric measures and oxygen consumption in human cohorts. (**A**) Association of Hb levels with BMI in NFBC1966 at 46 years. Unadjusted regression line (blue) with 95% CIs (gray) of blood Hb levels (g/liter) with BMI (kg/m^2^). (**B**) Forest plot representing the effect size estimates and their 95% CIs for 1 SD change in anthropometric measures per 1 SD change in Hb. Red, blue, and black lines indicate effect sizes for NFBC1966 at 46 years, YFS at 42 years, and meta-analysis, respectively. The effect sizes were adjusted for sex, smoking, physical activity, age, and height for parameters other than BMI. (**C** to **E**) Added variable plots for sex-adjusted partial associations of Hb levels with oxygen consumption at rest (C), oxygen consumption at rest with body fat percentage (D), and Hb levels with body fat percentage (E) in the NFBC1966 subpopulation (*n* = 123) at 31 years.

We then examined the association of Hb levels in humans with anthropometric and metabolic parameters in a cross-sectional and longitudinal design from 31 to 46 years in the general population-based, sea-level Northern Finland Birth Cohort 1966 (NFBC1966) (*11*, *12*) and searched for a replication in another sea-level cohort, Cardiovascular Risk in Young Finns Study (YFS) (*13*), at a mean age of 42 years (table S1 and fig. S1). After adjusting for potential confounders, we found a positive association between Hb levels and body mass index (BMI) in NFBC1966 at the age of 46 in both sexes (Fig. 3A and fig. S2A), and this association was replicated in YFS at 42 years [Fig. 3B and table S2, meta-analyzed effect size estimate (Beta) 0.27; 95% confidence interval (CI) 0.21 to 0.33; *P* = 2.2 × 10^−19^]. The increase of 10 g/liter in Hb corresponded to an increase of 3.5% in BMI, i.e., in an individual with a BMI of 25.0 kg/m^2^, this corresponds to a BMI increase of 25.9 kg/m^2^ (Fig. 3A and table S3). Hb levels were also positively associated with waist circumference, hip circumference, waist-hip ratio, body fat percentage, and visceral fat area, and negatively associated with body muscle mass ratio (Fig. 3B and table S2). Thus, the subjects with lower Hb levels appeared to be less abdominally obese and to have more muscle tissue. Similar associations were found for hematocrit levels and red blood cell counts with BMI as for Hb in males and females (table S4 and fig. S2, B and C). Also, the associations between Hb levels and the anthropometric parameters were similar in males and females (fig. S3). We found further positive associations between Hb levels and oxygen consumption at rest, Hb levels and body fat percentage and oxygen consumption at rest, and body fat percentage in a subpopulation of NFBC1966 (*n* = 123) (Fig. 3, C to E, and table S5), indicating that the individuals with lower Hb levels had less fat and consumed less oxygen.

### Lower Hb levels associate with healthier key metabolic measures in humans

**Fig. 4:**
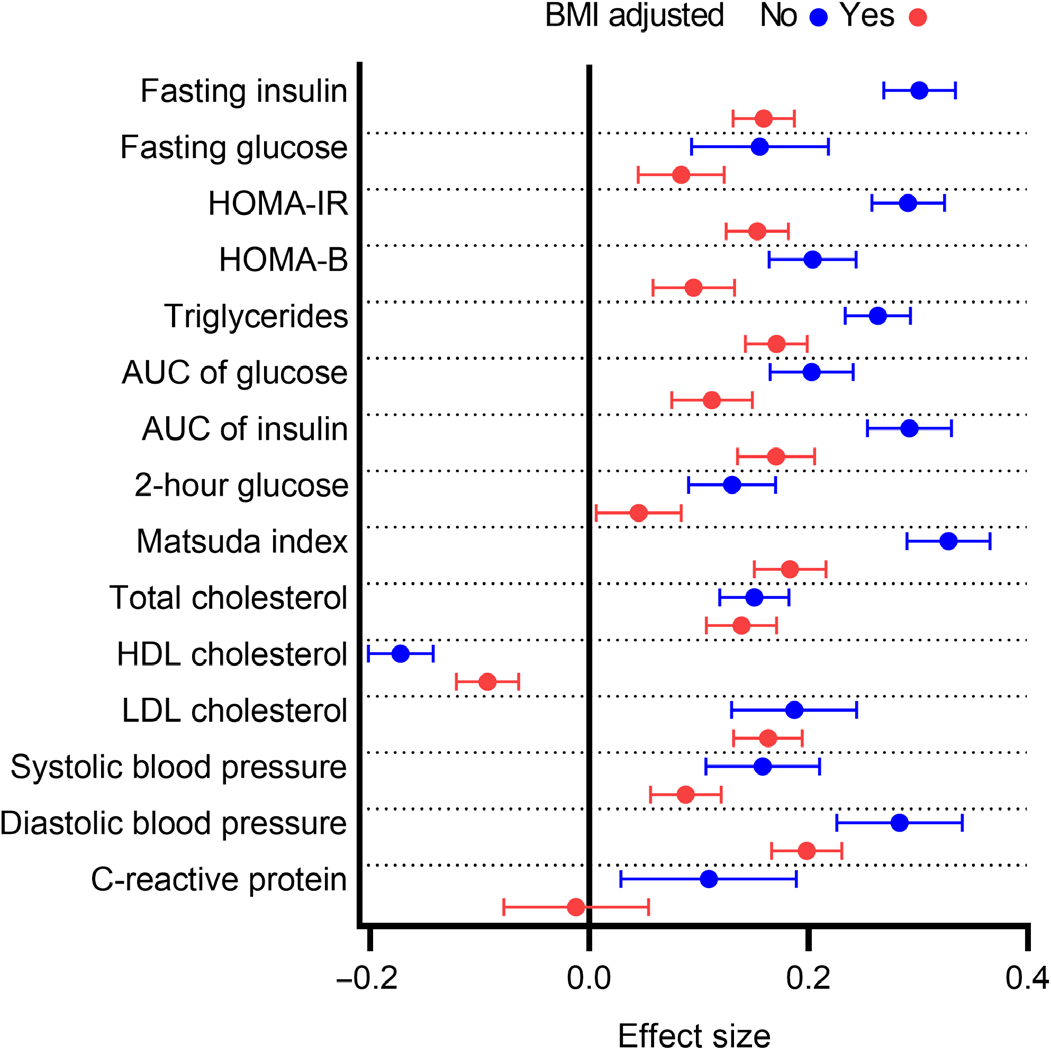
Association of Hb levels with key metabolic parameters. Forest plot representing the effect size estimates and their 95% CIs of the association in SD units of Hb levels with log(fasting insulin) and fasting glucose levels, log(HOMA-IR) and log(HOMA-B) indexes, log(triglycerides), log(AUC of glucose in OGTT)*, log(AUC of insulin in OGTT)* and 2-hour glucose in levels in a 2-hour OGTT*, log(Matsuda Index)*, fasting serum total cholesterol, HDL cholesterol and LDL cholesterol levels, systolic and diastolic blood pressure, and log[high-sensitivity C-reactive protein (CRP)] levels in meta-analysis of NFBC1966 at the age of 46 and YFS at 42 years, respectively. * indicates the associations only analyzed in NFBC1966. The effect sizes were adjusted for sex, smoking, physical activity (blue), and, in addition, BMI (red). Values with ±3 SD exclusions are presented.

Hb levels associated with fasting glucose and insulin levels, HOMA-IR and HOMA of β cell function (HOMA-β) indices, and area under the curve (AUC) of glucose and insulin in an oral GTT (OGTT) and the Matsuda Index in the meta-analyzed NFBC1966 at 46 years and YFS at 42 years, suggesting that subjects with lower Hb levels had better glucose tolerance and insulin sensitivity than those with higher Hb levels (Fig. 4 and table S6). Hb levels also associated positively with systolic blood pressure (SBP) and diastolic blood pressure, serum total cholesterol, low-density lipoprotein cholesterol (LDL-C), triglycerides (TG), and serum high-sensitivity (hs) C-reactive protein (CRP), respectively, and negatively with high-density lipoprotein cholesterol (HDL-C) levels (Fig. 4 and table S6). The effect sizes of these associations weakened when adjusted for BMI, but apart from hsCRP and 2-hour glucose, all associations remained below the Bonferroni-corrected threshold of *P* < 0.002 (Fig. 4 and table S6). When comparing the associations between males and females, the effect sizes were somewhat weaker for females; however, the directions of the associations were concordant throughout (fig. S4).

### Positive association of Hb levels with metabolic parameters strengthens with age

**Fig. 5:**
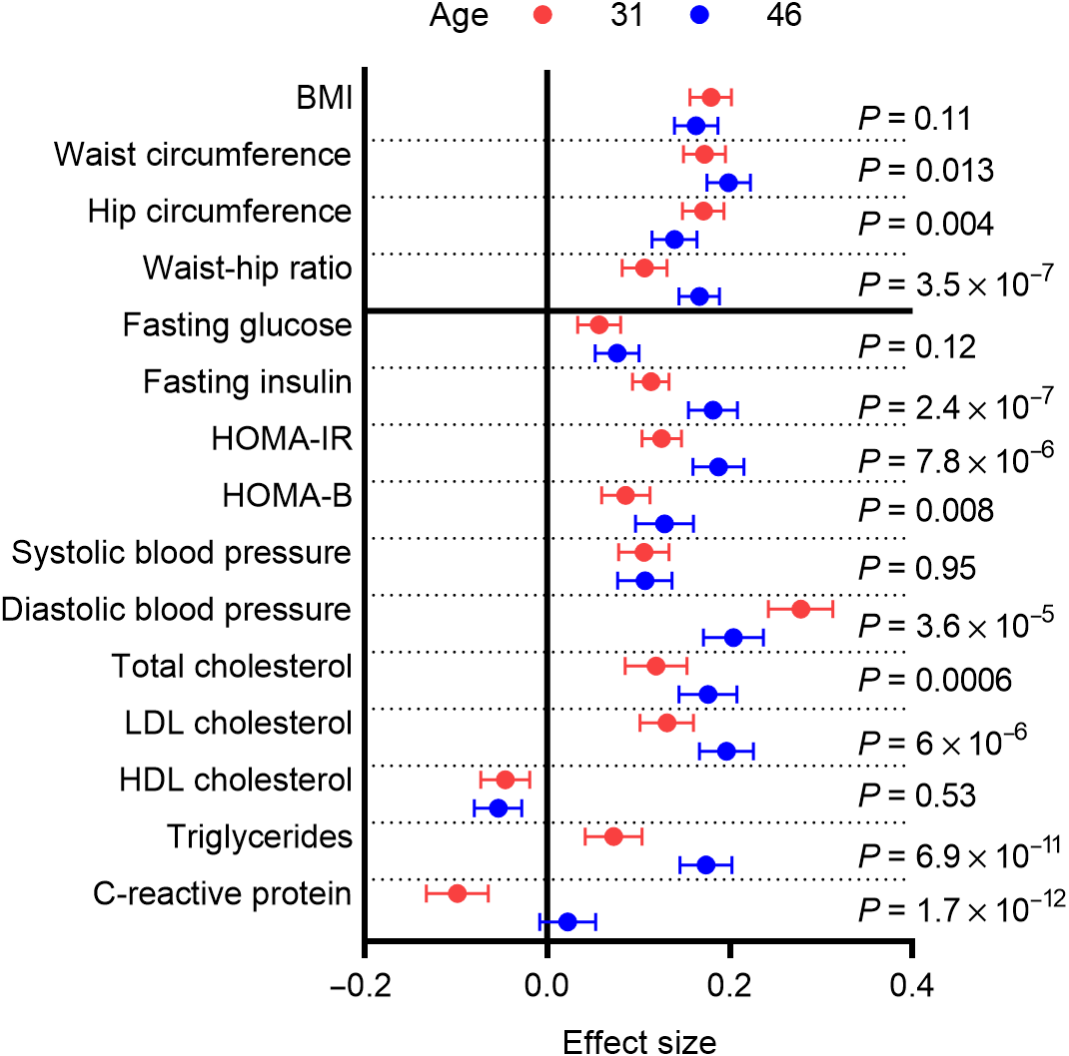
Positive association of Hb levels with most anthropometric and metabolic parameters strengthens with age. Effect sizes of association in SD units of Hb levels with log(BMI), waist and hip circumference, waist-hip ratio, fasting glucose and log(fasting insulin) levels, HOMA-IR and HOMA-B indexes, systolic and diastolic blood pressure, fasting serum cholesterol, LDL cholesterol, HDL cholesterol and triglyceride levels, and log(high-sensitivity CRP) in NFBC1966 at the age of 31 (red) and 46 (blue) years, respectively. The *P* values for the difference in effect size between age 31 and 46 years are indicated. The effect sizes for the anthropometric parameters (above the black horizontal line) were adjusted for sex, smoking, physical activity, and height (excluding BMI), and the metabolic parameters were adjusted for sex, smoking, physical activity, and BMI.

To evaluate the associations of Hb levels with anthropometric and metabolic measures in a longitudinal setting, we compared the effect sizes of these associations in subjects of NFBC1966 that had been examined both at the age of 31 and 46 years (*n* = 3624). The mean Hb levels remained unchanged from 31 to 46 years (fig. S5), and in general, the effect sizes of the associations increased with age or remained the same (Fig. 5), the most pronounced increases in effect sizes being found for the association of Hb levels with the levels of TG, fasting insulin, HOMA-IR, and waist-hip ratio, respectively (Fig. 5 and tables S7 and S8).

### Lower Hb levels associate with less adverse metabolite profiles

**Fig. 6:**
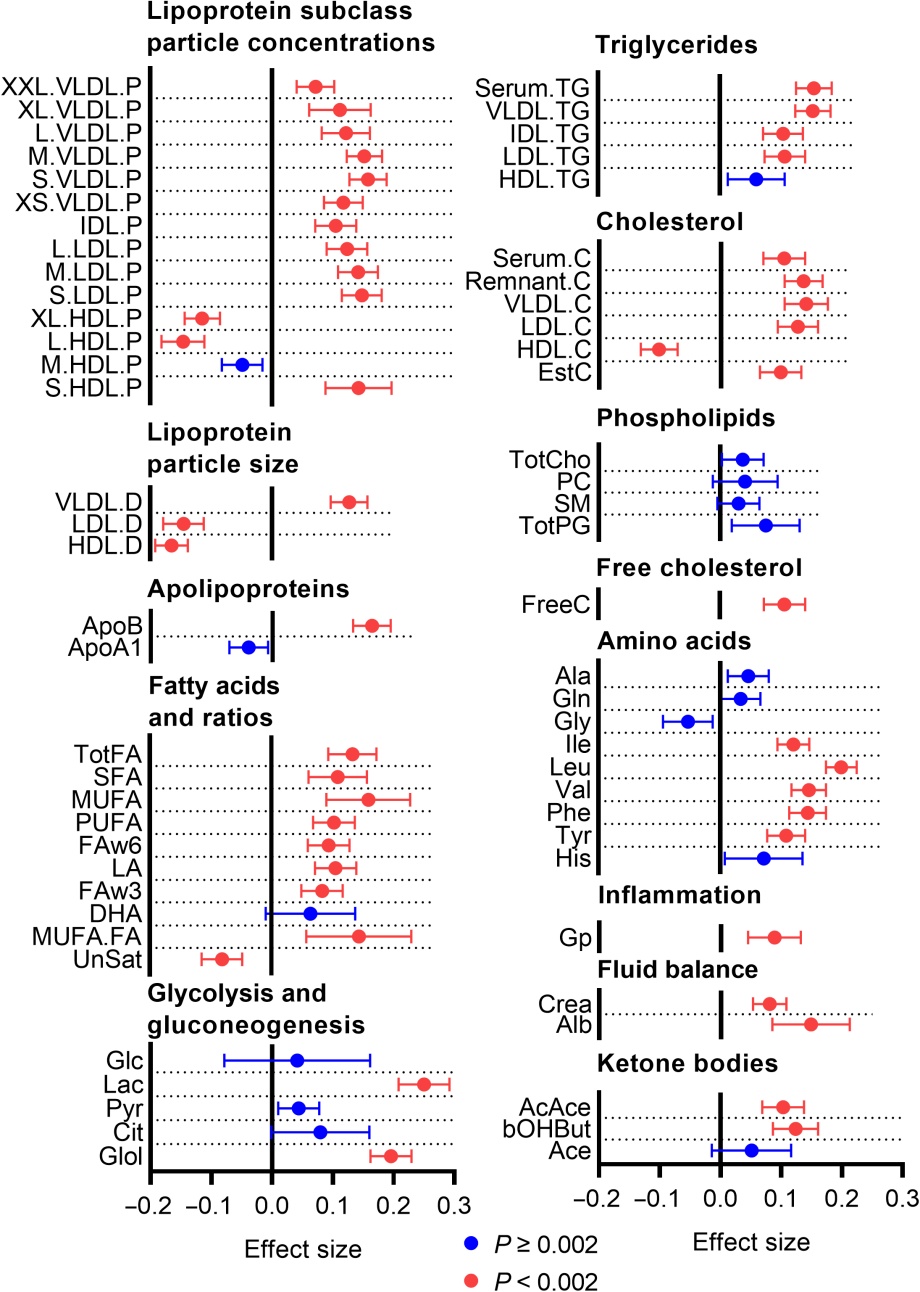
Higher Hb levels associate with metabolic signatures of adiposity, insulin resistance, and cardiovascular risk. Effect sizes in SD units of association of Hb levels with systemic metabolite levels in random-effects meta-analysis of NFBC1966 at 46 years and YFS at 42 years. The effect sizes were adjusted for BMI, sex, smoking, and physical activity.

We then evaluated the associations between Hb levels and systemic metabolite profiles of 150 variables analyzed by the nuclear magnetic resonance (NMR) metabolomics platform in the meta-analysis of NFBC1966 at 46 years and YFS at 42 years. Evidence for associations of Hb levels was found with lipoprotein subclass particle concentrations and particle sizes and with levels of ApoB, TG and cholesterol in lipoproteins, fatty acids (FAs), lactate, glycerol, branched-chain amino acids (isoleucine, leucine, and valine), aromatic amino acids (phenylalanine and tyrosine), the inflammatory glycoprotein acetyls, creatinine, albumin, and ketone bodies, respectively; these associations were mostly positive (Fig. 6 and table S9). We also examined whether longitudinal changes in the Hb-metabolite associations are similar to the cross-sectional changes. The magnitudes of cross-sectional and longitudinal effect estimates of the Hb level difference from 31 to 46 years on the NMR metabolite levels in NFBC1966 were consistent (fig. S6). The estimated slope for regression of longitudinal effects on cross-sectional effects was 0.97 (95% CI 0.89 to 1.05), indicating similar effect sizes for both the between-subject and the within-subject effects (fig. S6). Higher levels of branched-chain and aromatic amino acids, glycoprotein acetyls, and lactate, associated with the higher Hb levels here (Fig. 6 and table S9), have previously been associated with insulin resistance (*14*), and the first two have been associated with adiposity (*15*). Moreover, higher phenylalanine levels and higher ratio of monounsaturated FAs to all FAs, which associated with higher Hb levels here (Fig. 6 and table S9), have earlier been associated with increased risk for cardiovascular events (*16*).

### Both Hb levels and expression of HIF target genes associate with metabolic traits in causality analyses

**Fig. 7:**
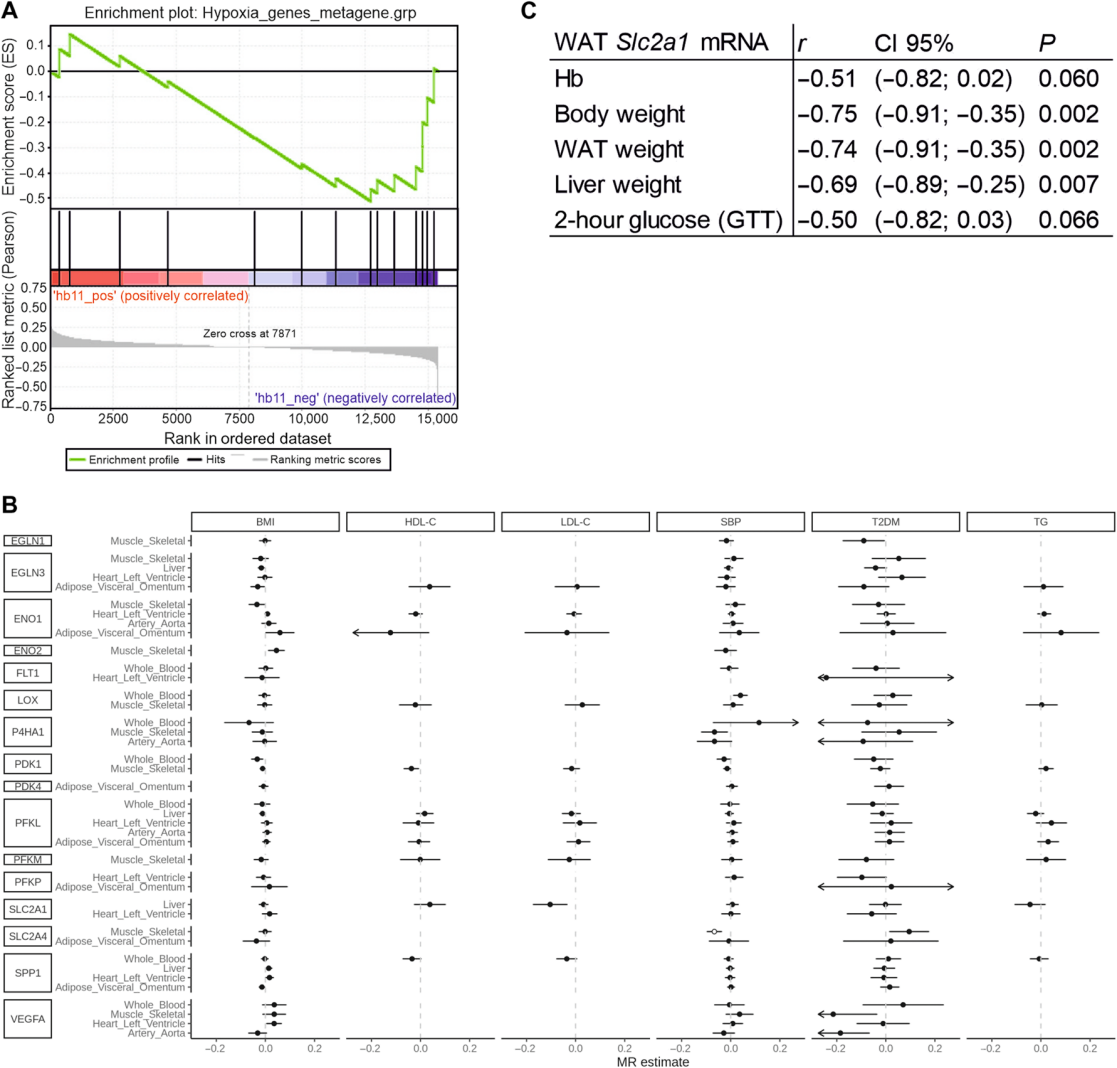
Genetic analyses between Hb levels and HIF target genes and metabolic outcomes. (**A**) GSEA of the YFS lowest Hb quartile (Hb < 132 g/liter, *n* = 392) versus YFS highest Hb quartile (Hb > 152 g/liter, *n* = 371) and selected hypoxia-induced HIF target genes. Cohort *n* = 1636. The analysis was adjusted for sex, age, numbers of thrombocytes and leukocytes, the first five principal components of the transcriptomics data, BMI, research center, and three technical microchip variables. (**B**) eQTL MR analyses of selected HIF target genes in the indicated tissues. The hollow point indicates *P* < 0.00125 (Bonferroni-corrected threshold of 0.05 for 40 exposures). *ENO1*, enolase 1; *FLT1*, VEGR receptor 1; *LOX*, lysyl oxidase; *P4HA1*, collagen prolyl 4-hydroxylase subunit alfa 1; *PDK1*, pyruvate dehydrogenase kinase 1; *PFKL*, phosphofructokinase liver; *PFKM*, PFK muscle; *PFKP*, PFK platelet; *SLC2A1*, glucose transporter 1; *SPP1*, secreted phosphoprotein 1; *VEGFA*, vascular endothelial growth factor A. (**C**) Association of WAT *Slc2a1* mRNA levels with key anthropometric and metabolic markers and Hb levels in 1-year-old C57Bl/6 male mice (*n* = 14).

We then carried out gene set enrichment analysis (GSEA) by associating Hb levels with expression of a set of HIF target genes reported to be induced at least fourfold by hypoxia in human peripheral blood monocytes (table S10) in whole-blood genome wide-expression data in a subpopulation of YFS (*n* = 1636). After adjustment for confounding factors, we found evidence for gene set level transcriptional activation of HIF target genes in individuals in the lowest Hb quartile (Hb < 132 g/liter) compared to the highest quartile (Hb > 152 g/liter) (Fig. 7A).

To evaluate the potential causal effect of Hb levels on the metabolic traits, we conducted MR analyses using genetic association estimates for Hb levels and cardiometabolic outcomes [BMI, HDL-C, LDL-C, SBP, type 2 diabetes (T2DM), and TG] from large-scale genome-wide association studies (table S11). There was no robust evidence for genetically proxied Hb levels being associated with any of the considered cardiometabolic traits (figs. S7 and S8). However, the point estimates for HDL-C, T2DM, and TG were consistent with our hypothesis (Fig. 1 and figs. S7 and S8). We next conducted MR using expression quantitative trait loci (eQTL) of HIF target genes as the instrumental variables. The results showed genetically proxied *SLC2A4* expression in skeletal muscle, coding for GLUT4, a major glucose transporter, being associated with SBP (Fig. 7B). As a further support, expression of *Slc2a1* mRNA, coding for GLUT1, in murine white adipose tissue (WAT) associated negatively with body weight and the weights of WAT and liver, and similar associations with glucose level at 2 hours after GTT and Hb were observed (Fig. 7C and table S12). Together, these data are consistent with the activation of the HIF response being beneficial on the metabolic profile.

### Manipulation of Hb levels alters metabolism in mice

**Fig. 8:**
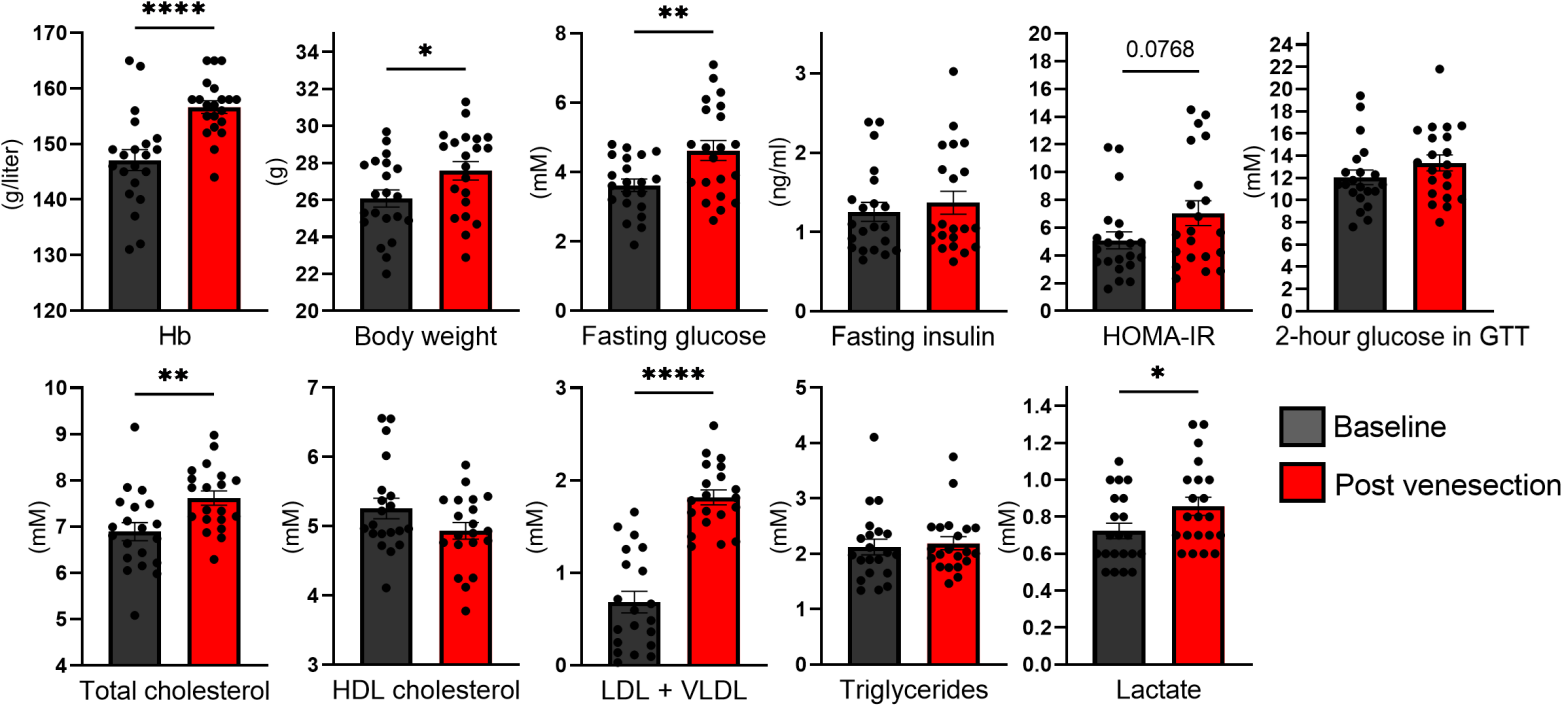
Manipulation of Hb levels by venesection in mice alters causally metabolic parameters. Hb levels, body weight, fasting blood glucose, fasting serum insulin, HOMA-IR, 2-hour blood glucose levels in GTT, total cholesterol, HDL cholesterol, LDL + VLDL cholesterol, triglycerides, and lactate levels of 3-month-old C57Bl/6 male mice (*n* = 21) before (Baseline) and 2 weeks after venesection (Post venesection). Data are means ± SEM. *P < 0.05, **P < 0.01, and ****P < 0.0001.

Last, we returned to the animal experiments and manipulated Hb levels in C57Bl/6 mice by venesection, which is followed by a physiological induction of erythropoiesis (*17*). Our data showed that, in 2 weeks, the venesection-induced erythropoiesis increased the Hb levels in mice and significantly increased body weight and fasting glucose, total cholesterol, LDL + VLDL cholesterol and lactate levels, with weaker evidence for an increased HOMA-IR score (*P* = 0.08) (Fig. 8). Association of the change in Hb levels with changes in metabolic parameters was concordant in effect sizes and consistent across analyses (fig. S9) and in agreement with those reported earlier for mice with genetically or pharmacologically activated HIF response (*9*, *18*). These data show that venesection was associated with systematic change in metabolic parameters, potentially due to change in Hb levels.

## DISCUSSION

The current study systematically investigated the associations of Hb levels with metabolism using observational, experimental, and causal analyses approaches. We found a positive association between Hb levels and BMI, independently of sex and age. Both males and females with the lower Hb levels had better glucose tolerance, lower total cholesterol and blood pressure levels, less adverse metabolite profiles, and lower inflammatory load. Notably, the observed associations were not only driven by BMI, and the effect sizes of many of them increased with age. Although standard MR analysis did not provide robust evidence to support a causal effect of Hb levels on cardiometabolic traits, the human eQTL MR and murine adipose mRNA expression level analyses indicated association of HIF target genes and metabolic regulators, *GLUTs 1* and *4*, with several key metabolic markers. Last, venesection in mice showed evidence for causal associations with body weight and metabolic parameters, potentially caused by the manipulation of Hb levels.

A few previous studies with limited sample sizes, selected cohorts, cross-sectional setting, and often one sex have shown associations of Hb levels with insulin resistance, hypertension, dyslipidemia, or metabolic syndrome (*19*–*23*), which are in line with our results. The underlying mechanisms have however been poorly understood. Hyperviscosity or changes in plasma volume, endothelial cell dysfunction, or higher iron/ferritin levels have been suggested as mediators of these associations. One study has reported a positive association between Hb concentration and incidence of metabolic syndrome in men (*24*), and another study has reported an increased risk for T2DM with higher hematocrit levels in men (*25*). The HIF pathway was only discovered after these early analyses (*6*). The current study is the first ever that has investigated whether the potential association of Hb levels with metabolism would be mediated by tissue oxygenation status involving activation of the HIF response by lower Hb levels (Fig. 1). Hb levels can be used as an indicator of tissue oxygenation status (*4*, *5*). The results presented here show that Hb levels associated with expression of HIF target genes. One of the key metabolic adjustments the hypoxia response confers is transcriptional reprogramming of energy metabolism (Fig. 1) (*6*, *7*). HIF target genes induce glucose intake insulin independently by up-regulating GLUTs and increase glucose usage in glycolysis while dampening the efficiency of the mitochondrial oxidative phosphorylation (*6*, *7*). While this mode consumes less oxygen, it also produces less adenosine triphosphate and can thus be considered more inefficient. Individuals being environmentally exposed to hypoxia by living at high altitude have been reported to have lower fasting glycemia and better glucose tolerance compared to those at near sea level and demographic studies associate living at high altitude with lower incidence of obesity and diabetes (*26*–*28*). Furthermore, in clinical trials for renal anemia, the first-in-class approved HIF-P4H inhibitor roxadustat, which stabilizes HIF and activates the hypoxia response pathway, reduced serum cholesterol and TG levels by ≥20% and lowered the LDL/HDL ratio (*29*).

The main strengths of this study are that while the previous studies have reported association of Hb levels with a single metabolic or cardiovascular health marker, we evaluated association of Hb levels with >170 anthropometric, metabolic, and inflammatory parameters including systemic metabolite profiles. These previous studies are mainly based on small and selected populations, while the current study is the largest study carried out in a nonselected birth cohort population (*n* = 5351), including a replication in another Finnish cohort (*n* = 1824; total *n* = 7175), and it has uniquely both cross-sectional and longitudinal design. The results were replicated in another vertebrate species. In addition, no previous studies have used genetic instruments for the assessment of these associations or studied metabolic effects of Hb manipulation in mice. The limitations of our study include the observational nature of human data and that we have not measured iron/ferritin levels, which are closely linked to Hb levels. However, the mean Hb levels remained stable for the 15-year period in the NFBC1966 cohort, suggesting that the low Hb levels were not anemic. Moreover, similar associations with BMI were seen between hematocrit levels and erythrocyte counts than Hb levels. One of the central questions in this study is the causality between the Hb levels and the metabolic outcomes. Since HIF is principally regulated posttranslationally at the protein level, the use of genetic instruments is not feasible and makes the analyses complicated. We have therefore used several methods to evaluate the causality of Hb/oxygen/HIF levels with the metabolic parameters, most of which but not all support the lower Hb/oxygen levels to drive the metabolic changes. We have used Hb levels in MR as an indirect proxy for oxygen in circulation at the population level and carried out lookups for relevant traits for eQTLs of HIF target genes. The limitation with the eQTL analyses is the concern whether the mRNAs are translated to proteins proportionally. We have also performed an intervention to manipulate Hb levels and studied its effects on metabolic parameters. Last, some of the metabolic HIF target genes can also be regulated with other transcriptions factors, such as MYC. However, this cooperation of MYC with HIF has mainly been reported to occur in cancer cells with high MYC levels, which is a very different setting from a young to middle age population cohort or wild-type mice studied here (*30*).

While more mechanistic studies are needed, our data are consistent with lower Hb levels contributing to metabolic and cardiovascular health by activating the HIF response that mediates the beneficial effects on metabolic health. Thus, the individuals with the lower endogenous Hb levels appear leaner and have a healthier metabolic and cardiovascular profile.

## MATERIALS AND METHODS

### Study design and setting

All animal experiments were carried out with wild-type C57Bl/6N mouse cohorts and performed according to protocols approved by the Provincial State Office of Southern Finland (license number ESAVI/6154/04.10.07/2014). The association of Hb levels with anthropometric and metabolic parameters in human was examined in cross-sectional and longitudinal design from 31 to 46 years in the sea-level general population-based NFBC1966 (*11*, *12*) (*n* = 3624 at 31 years and *n* = 5351 at 46 years), and a replication was performed in another sea-level cohort, YFS (*13*) (*n* = 1824) at 42 ± 5 years (referred as 42 years). The flow chart of the study and detailed characteristics of the human study populations are available in table S1 and fig. S1.

### Analyses in mice

Male C57Bl/6N mice (Charles River, Germany) were maintained in plastic cages at 21°C with a 12-hour light-dark cycle and free access to food. After weaning, the mice were fed Teklad Global 18% Protein Rodent Diet (18.6% protein, 6.2% fat, and 44.2% carbohydrates; Envigo); from 5 weeks of age, they were fed Teklad Global 19% Protein Extruded Rodent Diet (19.0% protein, 9.0% fat, 44.9% carbohydrates; Envigo) to slightly support increase in adipocity upon aging. At 11 months of age, the mice were subjected to a GTT performed after 12 hours of fasting under fentanyl/fluanisone and midazolam anesthesia. Mice were injected intraperitoneally with glucose (1 mg/g), and blood glucose concentrations were monitored with a glucometer. Serum insulin values were determined with Rat/Mouse Insulin ELISA kit (EZRMI-13K; Millipore), and HOMA-IR scores were calculated from the glucose and insulin values. The mice were weighed, sampled for Hb (HemoCue Hb 201), and euthanized at the age of 1 year. Total RNA was isolated from WAT with an E. Z. N. A. total RNA Kit II (Omega Bio-Tek) followed by reverse transcription with an iScript cDNA Synthesis Kit (Bio-Rad). Quantitative real-time polymerase chain reaction (qPCR) was performed with iTaq SYBR Green Supermix with ROX (Bio-Rad) in a C1000 Touch Thermal Cycler and a CFX96 Touch Real-Time PCR Detection System (Bio-Rad) with primer pairs 5′-AGAACAATCCAGACTAGCAGCA-3′ and 5′-GGGAACTTCACATCACAGCTC-3′ for *Tbp* mRNA and Quantitect primer assays (Qiagen) for *Slc2a1* mRNA.

For the venesection experiment, 3-month-old C57Bl/6N males fed Teklad Global 18% Protein Rodent Diet were fasted for 12 hours and anesthetized as above. Mice were weighted, and their fasting blood glucose and Hb levels and lactate levels were determined with a lactometer (Lactate Scout^+^ -meter, SensLab/EKF Diagnostics). Serum samples were collected for determination of fasting insulin and total cholesterol, HDL cholesterol, and TG levels by an enzymatic method (Roche Diagnostics). The LDL + VLDL levels were calculated from the total cholesterol, HDL cholesterol, and TG levels using the Friedewald formula (total cholesterol − HDL − (TG/2.2). GTT was performed with glucose (2 mg/g), and blood glucose concentration at 2 hours was monitored. The mice were then subjected to venesection of 0.2 ml (~20% blood volume). After 14 days, the same analyses (body weight, Hb, fasting glucose, fasting insulin, lactate, GTT, total cholesterol, HDL cholesterol, and TG) were carried out and the mice were euthanized.

### Human study populations and outcomes

The study conformed to the principles of the Declaration of Helsinki. The participants took part on a voluntary basis and signed their informed consent. The data were handled on a group level only, and personal information was replaced by identity codes. The research protocol was approved by the Ethics Committee of Northern Ostrobothnia Hospital District. NFBC1966 participants answered postal questionnaires and went through clinical examinations at 31 and 46 years (table S1). Body weight, height, and waist and hip circumference were measured at 31 and 46 years, and bioimpedance (body fat mass, fat percentage, muscle mass, and visceral fat area) was measured at 46 years. Fasting blood samples were taken at 31 and 46 years, and Hb, serum cholesterol (total, HDL and LDL), TG, glucose, insulin, and hsCRP levels and NMR metabolomics (150 metabolites) (*31*) were analyzed. In addition, hematocrit and red blood cell counts were analyzed at 46 years, and a 2-hour OGTT was performed at 46 years with insulin and glucose measurements at baseline and 30, 60, and 120 min after 75-g glucose intake. To evaluate the insulin resistance and β cell function, HOMA-IR and HOMA-β and Matsuda indices (*32*) were calculated based on OGTT insulin and glucose data. Brachial systolic and diastolic blood pressure was measured at 31 and 46 years. At 31 years, resting-state oxygen consumption measurement was performed for an NFBC1966 subpopulation (*n* = 123) at 31 years. NFBC1966 participants reported their smoking habits and physical activity at 31 and 46 years, and these were used as confounders in the analyses. Individuals using combined oral contraceptives, hormone replacement therapy, or serum lipid–lowering drugs were omitted from the metabolomics analyses, as these have been shown to greatly influence these measures (table S9).

The YFS participants were examined at the mean age of 42 years for body weight, height, and waist and hip circumference, and fasting blood samples were taken for analyses of Hb, serum cholesterol (total, HDL and LDL), TG, glucose, insulin and hsCRP levels, and NMR metabolomics (150 metabolites). YFS participants also reported their smoking habits and physical activity (*13*).

### Statistical analyses

In the mouse experiments, Student’s *t* test was used for statistical analyses for comparison of the metabolic traits between groups. Pearson’s correlation coefficient was calculated to compare linear dependences between *Slc2a1* mRNA level and each metabolic trait. AUC for GTT were calculated by the summary measures method.

In human cohorts, regression models were used to assess the associations between Hb (main explanatory variable) and anthropometric and metabolic variables (outcome). To adjust for potential confounding factors, smoking, physical activity, and sex were included as covariates in the regression models. For the analyses using YFS data, age was also included as a covariate. The analyses were conducted including or excluding observations with an absolute value more than three SDs off from the mean of Hb or the (potentially transformed) outcome variable. To ease the comparison of magnitudes of association across outcomes, the effect size estimates are reported in SD units. Of the anthropometric measures, BMI was log-transformed (to eliminate skewness and heteroscedasticity in model residuals). Height was included as an additional covariate in the regression models on anthropometric measures except in the model for BMI. Similar regression models were conducted for red blood cell counts and hematocrit in addition to Hb, to examine the association between these measures and anthropometric variables. Of the metabolic parameters, fasting insulin levels, Matsuda and HOMA indices, OGTT AUC of glucose and insulin, and TG and hsCRP levels were log-transformed to ensure symmetry and homoscedasticity of model residuals. In the hsCRP variable, including zeros in the values, the transformation used was log(*x* + *c*/2), where *c* = min (*x*_*x* ≠ 0_), i.e., the smallest nonzero value in the variable. Of the NMR metabolites, cube root transformation was applied to 144 variables that were measured in concentrations. The rest were left untransformed, except for the degree of unsaturation, which was log-transformed to ensure homoscedasticity in model residuals. The analyses were first conducted separately for NFBC1966 at 46 years and YFS at 42 years and then meta-analyzed for those outcomes available in both cohorts using an inverse-variance weighted (IVW) random-effects model. The meta-analysis effect sizes were calculated and reported also in the units in which they were measured. To evaluate whether the associations are driven by BMI, all analyses were repeated using BMI as an additional explanatory variable. To account for multiple comparisons, principal components analysis was used to estimate the number of independent tests for a Bonferroni-style multiple testing correction. As 23 principal components explained 95% of the variation in the anthropometric and metabolic outcomes, a *P* value of 0.002 (0.05/23) was used as a nominal threshold for significance in these analyses. R 3.3.2 software was used for all statistical analyses for human samples [R Core Team (2013) www.R-project.org/].

### Gene set enrichment analysis

To examine the hypothesized activation of HIF pathway, we performed GSEA (*33*) for a subset of YFS participants at 34 to 49 years (*n* = 1636) with Hb levels and whole-blood genome-wide expression profiling data (*34*). The tested pathway included 22 selected HIF target genes as justified in table S10. The analysis was done adjusted for age, sex, BMI, YFS study center, leukocyte and thrombocyte counts, five first principal components of transcriptomics data, and three technical variables from microarray hybridizations (chip, plate, and well). The analysis was done with residual gene expression profiles calculated by linear regression of each gene expression probe and abovementioned variables. A nominal *P* < 0.05 was considered significant.

### Mendelian randomization

We conducted MR to investigate the potential causality of Hb levels on cardiometabolic traits. In MR, genetic variants, randomly allocated at conception, are used as unconfounded instrumental variables, proxies, to study the effect of an exposure on an outcome. Genetic association estimates for Hb levels were taken from a genome-wide association study (GWAS) meta-analysis performed using the INTERVAL study, UK Biobank, and UK BiLEVE (a subset of the UK Biobank cohort), which included a total of 173,480 subjects of European ancestry (*35*). Adjustments were made for age, sex, BMI, alcohol consumption, and smoking status. For included UK Biobank participants, the SD of Hb levels was 0.95 g/dl in males and 0.89 mg/dl in females. Instruments were selected as variants that associated with Hb concentration at genome-wide significance (*P* < 5 × 10^−8^) after clumping to linkage disequilibrium *r*^2^ < 0.001 using the TwoSampleMR package of R (*36*).

To investigate whether similar results were obtained when using Hb genetic association estimates that did not adjust for BMI, alcohol consumption, and smoking status, we performed sensitivity analyses selecting instruments and genetic association estimates from a GWAS on Hb levels in 361,194 UK Biobank participants of European descent, with adjustment made for 20 principal components of genetic ancestry, age, age^2^, sex, age × sex, and age^2^ × sex (Neale Lab accessed 16 June 2020; rapid GWAS of thousands of phenotypes in the UK Biobank 2020 www.nealelab.is/uk-biobank/ukbround2announcement). Genetic association estimates for outcomes (BMI, LDL-C, HDL-C, TG, SBP, and T2DM) were taken from the published studies detailed in table S11 (*37*–*40*).

The main MR analysis estimating the association of genetically proxied Hb levels with cardiometabolic traits was performed using the IVW method (random-effects model) (*41*). This method combines the causal effect estimates from each individual genetic variant (calculated as the ratio of the variant–cardiometabolic trait association with the variant–Hb concentration association). It provides a reliable estimate if all genetic variants are valid instrumental variables (*42*). Sensitivity analyses that relax their assumptions on associations of the genetic variants with cardiometabolic traits through pathways independent of Hb levels were performed to explore the robustness of the findings. Specifically, we performed the weighted median method (*43*) and the Egger method (*44*). Both of these methods provide a statistically consistent estimator of the true causal effect under different sets of assumptions. The weighted median provides robust causal effect estimates when more than half of the weights of genetic variants is provided by valid instruments. The Egger method allows all genetic variants to be invalid but requires the pleiotropic effects that relate to the cardiometabolic outcomes through pathways to be uncorrelated with the genetic variant-Hb associations, and is also sensitive to outliers. The intercept test of the Egger method was used to test for unbalanced pleiotropy. Causal effect estimates are expressed per SD increase in genetically predicted Hb levels.

We also conducted MR to evaluate the effect of expression of HIF target genes on the same cardiometabolic traits. The *cis*-eQTL (within ±250 kb of the gene) associations were extracted from the GTEx v8 database (*45*), filtering them by *P* < 5 × 10^−4^ and clumping them at *r*^2^ < 0.1. The same outcome genetic association estimates were used as above. The MR effect size was estimated using the Wald ratio, and its standard error was calculated using the Delta method. All MR analyses were performed with the MendelianRandomization package in R (*46*).

## SUPPLEMENTARY MATERIALS

Supplementary material for this article is available at http://advances.sciencemag.org/cgi/content/full/7/29/eabi4822/DC1

## Appendix

View/request a protocol for this paper from *Bio-protocol*.
